# Exploring the Involvement of PINK1 in Parkinson’s Disease: A Scanning Tunnelling Microscopy Study of Electron Transfer in Synthetic DNA Samples

**DOI:** 10.21315/mjms-12-2024-956

**Published:** 2025-08-30

**Authors:** Muhammad Hanif Che Lah, Mohammed Faruque Reza, Shaharum Shamsuddin, Isao Watanabe, Jafri Malin Abdullah

**Affiliations:** 1 Department of Neurosciences, School of Medical Sciences, Health Campus, Universiti Sains Malaysia, Kelantan, Malaysia; 2 School of Health Sciences, Health Campus, Universiti Sains Malaysia, Kelantan, Malaysia; 3 USM-RIKEN Interdisciplinary Collaboration for Advanced Sciences (URICAS), Universiti Sains Malaysia, Pulau Pinang, Malaysia; 4 Nuclear Structure Research Laboratory, RIKEN Nishina Center, RIKEN, Saitama, Japan; 5 Brain and Behaviour Cluster, School of Medical Sciences, Health Campus, Universiti Sains Malaysia, Kelantan, Malaysia

**Keywords:** Parkinson’s disease, PINK1, DNA, electron transfer, scanning tunnelling microscopy, voltage gap

## Abstract

**Background:**

Parkinson’s disease (PD) is a neurodegenerative disorder with a complex aetiology involving several genetic and environmental factors. Although no clear evidence of a direct link between the electronic features of DNA and PD has been found, elucidating the role of DNA in cellular function and dysfunction could provide valuable insights into the mechanisms of the disease (e.g. mutations occurring in the phosphatase and tensin homolog [PTEN]-induced kinase 1 [PINK1] DNA of PD). This study aimed to analyse topographic images and measure the electronic conductivity of synthetic normal and mutant PINK1 DNA molecules.

**Methods:**

Two 15-mer synthetic oligonucleotides of Oligo1 normal PINK1 (5′-CAG CTG CTG GAA GGC-3′) and Oligo2 mutant PINK1 (5′-CAG CTG CCG GAA GGC-3′) were measured using scanning tunnelling microscopy and spectroscopy.

**Results:**

The study’s findings revealed that the mean values of the voltage gap (V_g_) between Oligo1 normal and Oligo2 mutant PINK1 DNA molecules at the mutation region A2–C2 are 1.204 ± 0.198 V and 0.676 ± 0.495 V, respectively, indicating differences in the electronic properties between the Oligo1 normal and Oligo2 mutant PINK1 DNA molecules. However, the mean V_g_ values of Oligo1 normal and Oligo2 mutant PINK1 DNA molecules were found to not significantly differ from each other (*P* = 0.162 > *α* = 0.05).

**Conclusion:**

The study found that the voltage gap between normal and mutant PINK1 DNA molecules is not significantly different, suggesting that DNA sequence differences may not directly alter electrical properties. However, PINK1 mutations play a role in early-onset PD due to mitochondrial dysfunction, and future therapies should focus on restoring PINK1-Parkin signalling and mitochondrial health.

## Introduction

Parkinson’s disease (PD), the most common neurodegenerative disorder after Alzheimer’s disease (1, 2), presents a complex set of challenges that include a variety of motor and nonmotor symptoms (3–5). Currently, there is no cure for PD, and PD treatment focuses primarily on symptom relief carried out using medications such as levodopa. PD affects approximately 1%–2% of the population over the age of 50, with an estimated 1.5 million cases occurring in the United States of America (USA) alone; moreover, PD imposes considerable financial and emotional costs to patients and their caregivers (1, 6). The Global Burden of Disease 2019 provides statistics on the burden of PD, indicating that the disease’s prevalence has increased in 204 countries and territories worldwide between 1990 and 2019 (7–9). Over-65 age groups had the highest number of PD patients, and the proportion of patients over the age of 80 increased significantly over the same period, particularly in the USA and Norway (7, 10).

Genetic variations associated with PD have been increasingly found as a significant risk factor for the Southeast Asian population, particularly mutations in the leucine-rich repeat kinase 2 (LRRK2) gene, such as G2385R and R1628P, which are prevalent in East Asian communities, especially among Chinese and Japanese individuals (11–13). G2385R and R1628P polymorphisms have been associated with a higher incidence of PD in both Malay and Chinese ethnic groups in Malaysia (11, 14). The incidence of PD in Malays – particularly phosphatase and tensin homolog (PTEN)-induced kinase 1 (PINK1), in which the amino acid leucine is replaced by proline (p.Leu347Pro) – is 6.9% in individuals who develop the disease at or before the age of 50 years (15). The p.Leu347Pro mutation is more common in Malays than in other ethnic groups, although some cases have been reported in the Indian population (11, 16, 17). The association between the mutation of the PINK1 gene and the increased incidence of PD in certain ethnic groups plays an important role in understanding the genetic basis of early-onset PD.

Scanning tunnelling microscopy (STM) is a powerful technique that enables the direct observation of individual biomolecules at the molecular level and has been used to make important contributions to physics as well as surface and materials science. STM is particularly important for revealing the intricate structures of biological molecules and exploring fundamental interactions at the nanoscale, leading to insights that can impact various fields of study, such as biophysics and biomaterials (18). This technique utilises quantum tunnelling to allow researchers to study the electronic properties and topography of surfaces at near-atomic resolution (19).

Despite advances in STM technology, STM’s use in understanding the electron transfer properties of DNA in the context of PD remains relatively unexplored, providing an opportunity for the mechanisms of electron transfer to be elucidated to better understand the biological processes involved in DNA damage and repair associated with PD. Given the recognised role of DNA damage in neurodegeneration, particularly in PD (2, 5, 11, 14), studying the topographic and electronic properties using STM could provide important insights on the disease’s molecular basis. Hence, to address the above-mentioned gap, this study aimed to acquire topographic STM images and measure the electronic conductivity of normal and mutant PINK1 DNA molecules.

## Methods

STM was used to study electron transport in synthetic single-stranded normal and mutant PINK1 DNA molecules. Both DNA samples were measured using STM and scanning tunnelling spectroscopy (STS) to obtain topographic images and record the current–voltage (I–V) curves, respectively. I–V spectroscopy measurements were used to analyse the electronic conductivity in the DNA and determine the voltage gap associated with electron transfer mechanisms. The DNA samples were prepared for STM measurements using a modified version of the method used in the works of Jin et al. (20) and Arscott and Bloomfield (21).

### Sample Preparation

**Figure 1:**
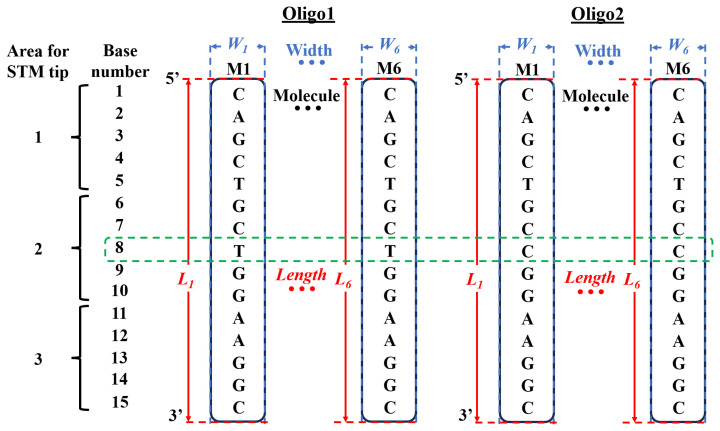
Schematic representation of the DNA sequences, the length and the width of the molecules of the Oligo1 normal and Oligo2 mutant PINK1, showing the difference of the oligonucleotides T and C, at base number 8. Area 1 was roughly estimated for base numbers 1–5 of the DNA sequences, Area 2 for 6–10 and Area 3 for 11–15. The length and width were measured on six different molecules of each DNA sample. A = adenine; C = cytosine; G = guanine; *L_x_* = length; M_x_ = molecule; T = thymine; *W_x_* = width

Two 15-mer single-stranded synthetic oligonucleotides of Oligo1 normal (5′-CAG CTG CTG GAA GGC-3′) and Oligo2 mutant (5′-CAG CTG CCG GAA GGC-3′) DNA sequences of PINK1 (Integrated DNA Technology, IDT, Singapore) were selected based on Tan et al.’s study (15). The reported PINK1 p.Leu347Pro mutation is particularly notable due to its higher prevalence in Malays compared to other ethnic groups. The 15-mer sequences were designed to represent the normal and mutant variants around the mutation site with a single nucleotide difference at the eighth position to enable the investigation of mutation-specific electronic conductivity using STM. The difference between Oligo1 and Oligo2 lies in the eighth position of the base number of the DNA sequences, where thymine (T) and cytosine (C) differ (Figure 1). According to the IDT FirstBASE technical bulletin, the synthesised standard desalted oligonucleotides typically achieve a purity of about 75% due to having a coupling efficiency of about 99.25% (22).

The samples were prepared by dissolving a small amount (~0.01 mg) of DNA in 1.5 mL of pure water. Type I pure water was prepared and purified by the host laboratory (Department of Applied Physics, Faculty of Engineering, Hokkaido University, Sapporo, Hokkaido, Japan) to avoid contamination during the preparation of the DNA solutions (23–25). Moreover, the sample solutions were sonicated in an ice-water bath for 60 minutes (26–29) to prevent the DNA from thermally degrading. An ultrasonic cleaner (Branson Model 1510 Ultrasonic Cleaner, Branson Ultrasonics Corporation, Connecticut, USA) was used to homogenise the oligonucleotide molecules and prevent the molecular structure from tangling and sticking together.

### Preparation of the Flat Surface of the Highly Oriented Pyrolytic Graphite (HOPG) Substrate

HOPG (Bruker Corporation, Billerica, Massachusetts, USA) was chosen as the substrate because its flat surface is highly reproducible, allowing the substrate to be easily prepared for the deposition of the samples. HOPG is also inexpensive when used as a substrate material (30–32); for example, HOPG is often used as a substrate for STM to study biomolecules such as DNA (18, 20, 30). Furthermore, HOPG has atomically smooth surfaces that are well suited for STM applications. These surfaces enable high-resolution imaging and analysis, making them particularly useful for the study of DNA molecules. The stability and conductive properties of HOPG make it even more suitable for this study.

**Figure 2:**
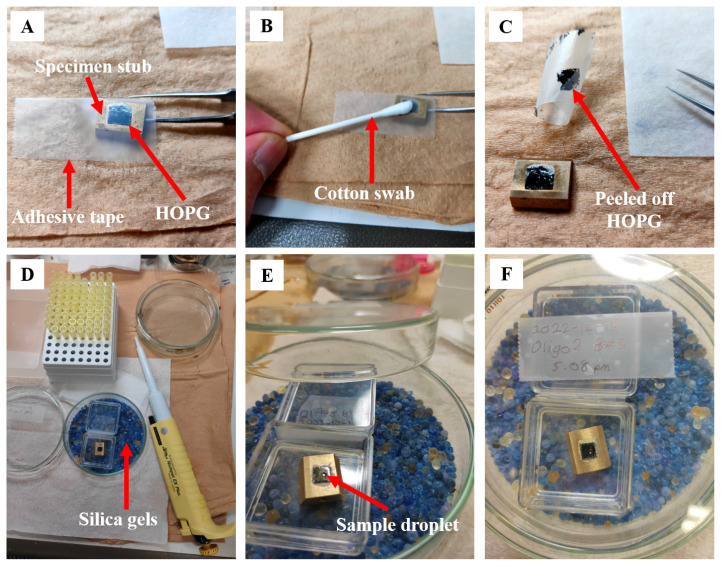
Preparation of DNA samples for STM measurement: cleavage of the fresh HOPG surface (A–C) and drying of the sample droplet overnight (D–F). (A) Adhesive tape is applied to the surface of the 10 mm x 10 mm HOPG attached to the specimen stub; (B) Gentle pressure is applied to the tape with a cotton swab; (C) The tape is peeled off to reveal a freshly cleaved HOPG surface; (D) Deposition of the sample on freshly cleaved HOPG; (E) Evaporation of the sample droplet in the air; (F) Sample on the specimen stub after drying overnight in silica gels, ready for STM measurement. HOPG = highly oriented pyrolytic graphite; STM = scanning tunnelling microscopy

HOPG with a size of 10 mm × 10 mm was attached to the surface of a gold-plated brass specimen stub via silver paste to enhance the electrical conductivity of the mounted samples (Figure 2A) (33). An epoxy adhesive (Araldite, Huntsman Corporation, Texas, USA) was used to improve the mechanical attachment of the HOPG to the brass stub. The specimen stub acts as a platform holding the sample securely in place during the STM measurement while providing an electrical connection to apply a bias voltage between the sample and the STM tip, which is required for measuring tunnelling current. Gold is highly conductive and enables efficient electrical contact, while brass provides mechanical strength, stability and electrical conductivity.

The flat HOPG surface was freshly cleaved using the adhesive tape technique each time before sample deposition (Figures 2A–2C) (34–36). 3M 810-B Scotch Magic Tape (3M, Hutchinson, Minnesota, USA) was used to peel off the uneven graphite layer and create a flat surface. A strip of tape was applied to the surface and spread evenly over the HOPG surface by applying light pressure with a cotton swab (Figure 2B). After application, the tape was slowly and carefully peeled off to reveal a freshly cleaved, flat HOPG surface (Figure 2C).

### Sample Deposition onto HOPG

A total of 20 μL of the sample solution was applied to a freshly cleaved HOPG using a micropipette, as shown in Figure 2D. The water in the sample was evaporated in air at room temperature (37, 38) and then dried overnight to completely remove the water from the DNA samples (typically for more than 12 hours before STM measurement) in a closed Petri dish with moisture-absorbing silica gel as a desiccant (Figures 2E–2F) (39).

### STM and STS Measurements

STM measurements were performed with a JEOLSPM 5200 (JEOLSPM Ltd., Tokyo, Japan) using a sharp platinum-iridium (Pt/ Ir) tip under typical atmospheric conditions at room temperature in constant current mode at 0.100 nA and a bias voltage of −1.200 V to obtain topographic images of Oligo1 normal and Oligo2 mutant PINK1 DNA molecules. The Pt/Ir tip (Bruker Corporation, Billerica, Massachusetts, USA) was used due to the excellent mechanical and electrical properties of the alloy, which are crucial for high-resolution imaging in STM experiments (40, 41). In cases where in the tip became blunt or deformed due to impact or contact with the sample surface, which resulted in highly noisy images during data acquisition, the tip was carefully cut off to create a sharp tip, ensuring higher sensitivity and precision in detecting tunnel current fluctuations between the tip and the sample surface (42), as the atomic sharpness of the tip is crucial for high-resolution STM images.

**Figure 3:**
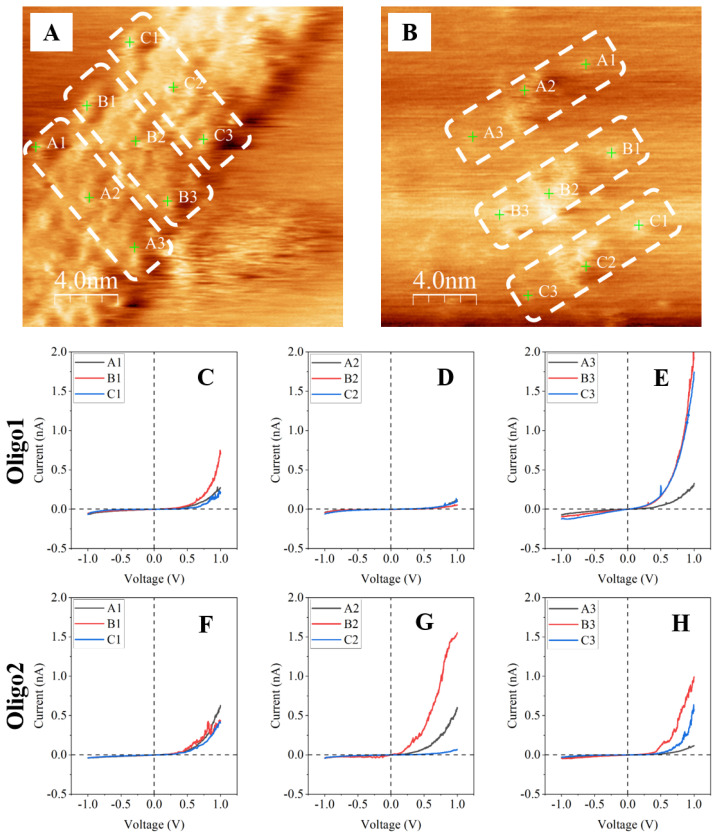
STS measurements were performed at the positions of the green markers on the Oligo1 normal (A) and Oligo2 mutant (B) PINK1 DNA molecules and the corresponding I–V curves (C-H). The bias voltage, V and feedback current, I for the STS were set to V = 1.000 V and I = 0.100 nA and the voltage measurement points were between −1.000 and 1.000 V. A1, B1 and C1 correspond to Area 1 in Figure 1; A2, B2 and C2 correspond to Area 2; A3, B3 and C3 correspond to Area 3. The STM tip was positioned in these areas to measure the I–V curves. The dashed lines in (A) and (B) roughly show the individual DNA molecules spreading across the HOPG surface due to the weak molecular interaction, i.e. van der Waals force. HOPG = highly oriented pyrolytic graphite; I–V = current–voltage; STS = scanning tunnelling spectroscopy

STS is used to detect current–voltage (I–V) characteristics of the sample in the STM by measuring the transverse conductivity of the molecule, which determines the electronic density of the states of the investigated samples (43–46). The sample’s I–V characteristics provide essential insights into the transport properties of materials by illustrating how tunnelling current varies with the applied voltage. STS measurements were performed on the Oligo1 normal and Oligo2 mutant PINK1 DNA samples at room temperature and ambient pressure according to the method used by Shapir et al. (44), albeit with some parameters modified (Figures 1, 3A and 3B). The bias voltage (V) and feedback current (I) parameters for the STS were set to V = 1.000 V and I = 0.100 nA. The parameters for the tunnelling spectra on the DNA samples were chosen to achieve the highest possible I–V quality in this system while avoiding possible damage to the molecules. Each I–V curve consisted of 1,024 measurement points with voltage values between −1.000 and 1.000 V averaged over all 32 points.

The I–V measurements were recorded on the Oligo1 normal and Oligo2 mutant PINK1 DNA molecules in three different areas (Figure 1). Area 1 was roughly estimated for base numbers 1–5 of the DNA sequences, Area 2 for base numbers 6–10 and Area 3 for base numbers 11–15. Each area was recorded three times on three different DNA molecules of Oligo1 and Oligo2, so that the total number of I–V measurements for each area was nine (*n* = 9). Since it was nearly impossible to precisely align the STM tip to the position of the different individual bases of the 15-mer long DNA sequences, Area 2 was hypothesised to be the area where the gross targeted difference lies. Three different areas of Oligo1 and Oligo2 were measured to ascertain whether a difference in the I–V characteristics can be detected in Area 2, where the difference in DNA sequences between T and C lies in the eighth base number. Areas 1 and 3 served as controls for the I–V measurement, as the DNA sequences in both areas are identical. The voltage gap was determined from the I–V curve by identifying the voltage range in which the tunnelling current is essentially zero or very low before significant current begins to flow (47, 48). This onset of current flow corresponded to the excitation of electrons across the energy gap of the sample and thus reflected the band gap or voltage gap of the material at the measurement location.

### Software for Data Acquisition, STM Image Analysis and Molecular Modelling

Data acquisition of the topographic STM images and the STS was performed using proprietary WinSPM Data Processing software (version 2.15, R. B. Leane, JEOL Ltd., Tokyo, Japan). WSxM 4.0 Beta 9.3, a freeware scanning probe microscopy software, was used to analyse the length profile of the topographic STM images (49). Avogadro (version 1.2.0, http://avogadro.cc/), an open-source tool for creating and visualising molecules, was used to build and estimate the length of the DNA molecules (50).

### Statistical Analysis

The statistical analysis in the current study was performed using OriginPro 2022 version 9.9.0.225 (OriginLab Corporation, Massachusetts, USA). Descriptive statistics of the length measurements of Oligo1 normal and Oligo2 mutant PINK1 DNA were determined on six different molecules of each DNA sample (Figure 1) (51). The sample size of the voltage gap of Oligo1 normal (*n* = 9) and Oligo2 mutant (*n* = 9) PINK1 DNA molecules was relatively small and imposed certain limitations on statistical inference, raising the question of whether a parametric or a nonparametric statistical test was appropriate for the study’s small sample sizes (52, 53).

A suitable statistical test was selected for the comparison of the voltage gap of the two independent DNA samples based on the fulfilment of the key assumptions required for hypothesis testing: i) random sampling; ii) normal distribution; and iii) homogeneity of variance (51–54). The STM measurements in the first assumption were considered to be from a random sample of independent DNA molecules prepared and measured under identical experimental conditions. In the second assumption, the normality of the samples was tested using the Shapiro–Wilk test due to the test’s suitability for small sample sizes (51, 55, 56). In the third assumption, the *F*-test was applied to assess the homogeneity of variances between the two groups (57, 58). Once the randomness, normality and homogeneity of variances were confirmed, the parametric independent-sample *t*-test was selected to compare the differences in the means of the voltage gaps (54). This methodological approach ensures that the conclusions drawn from the statistical comparison are as reliable as possible despite the limited sample size.

## Results

### Length of Oligo1 and Oligo2 PINK1 DNA Molecules

The raw STM topographic images of Figures 3A and 3B were analysed for the length profile measurement (Figure 1) for Oligo1 normal and Oligo2 mutant PINK DNA molecules, respectively. Table 1 presents a comparative analysis of DNA molecule lengths L obtained via STM and molecular modelling with Avogadro software for the Oligo1 normal and Oligo2 mutant PINK1 DNA molecules. For Oligo1, the STM-measured lengths of six individual molecules yielded a mean value of 12.551 ± 0.304 nm, which was tightly clustered around the median of 12.679 nm, indicating relatively consistent measurements across replicates. In contrast, Oligo2 showed a significantly shorter mean STM-measured length of 8.531 ± 0.397 nm with a median of 8.570 nm. Interestingly, Avogadro modelling yielded very similar theoretical lengths for both Oligo1 and Oligo2 at 5.332 nm and 5.352 nm, respectively, reflecting the theoretical backbone length of each oligo strand without considering the possible conformational or surface interaction effects observed in STM. This significant discrepancy in length between STM and modelling, particularly for Oligo1, likely reflects the influence of molecular stretching, deposition orientation and surface adsorption on the lengths observed in STM measurements.

### Voltage Gaps of Oligo1 and Oligo2 PINK1 DNA

Figures 3C–3F show the individual I–V curves with the measured voltage gaps (V_g_) of Oligo1 normal and Oligo2 mutant PINK1 DNA molecules at points A1–C1, A2–C2 and A3–C3, respectively (Table 2). The V_g_ measurements for the Oligo1 normal and Oligo2 mutant PINK1 DNA molecules at points A1–C1, A2–C2 and A3–C3 indicate different profiles. For Oligo1, V_g_ was the highest at A2–C2 (median = 1.259 V; mean = 1.204 ± 0.198 V) and the lowest at A3–C3 (median = 0.252 V; mean = 0.341 ± 0.208 V). In Oligo2, V_g_ was relatively consistent, with less variability at A1–C1 (0.626 ± 0.094 V) but greater variability at A2–C2 (0.676 ± 0.495 V). Oligo2 showed a higher V_g_ at A3–C3 (median = 0.936 V) compared to Oligo1. These results emphasise the differences in electrical properties between normal and mutant PINK1 DNA molecules.

The measurements revealed notable differences in the V_g_ profiles of Oligo1 normal and Oligo2 mutant PINK1 DNA molecules. Oligo1 exhibited the highest V_g_ at A2–C2, with a consistent median and mean; moreover, the lowest V_g_ was found at A3–C3, indicating reduced electrical activity at this point. In contrast, Oligo2 showed a more consistent V_g_ at A1–C1 but greater variability at A2–C2, reflecting differences in molecular behaviour. At A3–C3, Oligo2 had a significantly higher median V_g_ compared to Oligo1, suggesting altered electrical properties in the mutant molecules. These variations suggest structural or functional differences between normal and mutant PINK1 DNA.

The Shapiro–Wilk test for normality of the V_g_ measured at the corresponding points (A1–C1, A2–C2 and A3–C3) on Oligo1 normal and Oligo2 mutant PINK1 DNA molecules shows that the V_g_ vary between points and between oligos, with Oligo1 having a higher mean V_g_ at A2 (1.203 V) compared to Oligo2 at the same point (0.676 V) (Table 3). The Shapiro–Wilk test yielded *P*-values above the significance threshold (*α* = 0.05) for all comparisons, meaning that none of the datasets deviated significantly from the normal distribution. Therefore, the null hypothesis (*H_0_*) of normality could not be rejected for any of the six datasets, supporting the assumption of normality in the subsequent parametric analysis of the V_g_.

Table 4 presents the results of the *F*-test for homogeneity of variance between the V_g_ of Oligo1 normal and Oligo2 mutant PINK1 DNA molecules measured at three positions (A1–C1, A2–C2, and A3–C3). The *F*-test results revealed the lack of any statistically significant differences in variance at any of the points. The calculated *F* values in all comparisons were less than the critical value (19.000) (57), and the corresponding *P*-values exceeded the significance threshold (*α* = 0.05), leading to the conclusion that the *H_0_* of equal variances could not be rejected. Thus, evidence suggesting that the V_g_ variances between Oligo1 and Oligo2 differ significantly is insufficient, supporting the assumption of homogeneity of variance required for the following parametric analysis.

The results of the independent-samples *t*-test (Table 5) reveal that the *t*-statistics of the mean values of the V_g_ of Oligo1 normal and Oligo2 mutant PINK1 DNA molecules at points A1–C1 (*t* = 0.828), A2–C2 (*t* = 1.712) and A3–C3 (*t* = −2.537) are smaller than the critical value of the *t*-table (*t_crit_* = 2.776) (54). The *P*-values at points A1–C1 (*P* = 0.454), A2–C2 (*P* = 0.162) and A3–C3 (*P* = 0.064) of the V_g_ mean difference of Oligo1 normal and Oligo2 mutant PINK1 DNA molecules are greater than the *α* = 0.05. Therefore, the *H_0_* of the equal means cannot be rejected, indicating the lack of evidence to conclude that the mean values of the V_g_ of Oligo1 normal and Oligo2 mutant PINK1 DNA molecules are significantly different.

## Discussion

When DNA molecules are spread out on a HOPG surface, the DNA length measured with STM often appears longer than the length theoretically estimated with three-dimensional (3D) molecular modelling software such as Avogadro. This discrepancy is caused by several factors related to the physical adsorption and conformations of the DNA, which are influenced by surface interactions with HOPG and kinetic trapping effects rather than an ideal B-form DNA structure. When DNA is deposited on a 2D surface such as HOPG, the DNA molecules undergo significant changes due to adsorption interactions, leading to conformational changes such as bending, looping and folding. Such structural patterns can cause the deposited DNA to appear longer when adsorbed on the HOPG surface. Adsorption on HOPG is often described as kinetic trapping – a process in which DNA molecules are trapped in metastable conformations during drying or incubation and are prevented from relaxing into their shortest possible length (59). In the drying phase, the projected conformation of the DNA molecules may not be maintained due to the lateral capillary forces that affect DNA during drying (60). Liu et al. (61) emphasised that linear and circular DNA can form different structural patterns, often resulting in elongated shapes upon adsorption. This suggests that the strength of the interaction between the DNA molecules and the HOPG surface, including the weak molecular interaction of the van der Waals force, plays a crucial role in determining the resulting shape and length of the DNA strands.

STS measures the local density of states (LDOS) at specific positions on a sample, providing spatially resolved information about electronic states as a function of energy (62). The current study investigated the electronic conductivity of synthetic single-stranded DNA of Oligo1 normal and Oligo2 mutant PINK1 molecules using STS. The study measured I–V characteristics at three specific points on each DNA molecule, revealing slight but significant differences in conductivity profiles between normal and mutant DNA sequences. Such measurements might provide insights into the fundamental properties of DNA and have implications for better understanding mutation-induced electronic effects in biomolecules (63).

I–V spectroscopy involves measuring the response of the tunnelling current as bias voltage is systematically varied across a specified range. I–V spectroscopy is typically conducted with the Pt/Ir tip positioned at a fixed distance above the surface as voltage is gradually changed without feedback mechanisms to prevent unwanted adjustments in the gap width. The resulting I–V curve illustrates the relationship between voltage and tunnelling current, providing insights into both filled and empty states, which is accomplished by obtaining I–V spectra at selected points during constant current STM image acquisition.

Voltage gap refers to the minimum voltage required for a device to conduct current significantly. In semiconductor devices, voltage gap is closely related to the band gap energy, which is the energy difference between valence bands and conduction bands. The band gap determines how much energy is required to excite an electron from the valence band to the conduction band, facilitating electrical conduction. The higher the voltage gap or energy gap, the less electrons transferred from the molecules to the tip and vice versa, indicating that the molecules’ conductivity characteristics or conductance are low and similar to those of the insulator. However, in this study, conductivity or differential conductance can still be observed, indicating that the DNA molecules’ conductivity characteristics are similar to those of the semiconductor.

Differences in conductance have been observed in investigations of the electronic properties of individual DNA bases (adenine A, cytosine C, guanine G and thymine T) using STM. These differences have primarily been attributed to the unique chemical structures and electron configurations of individual bases, which can significantly affect electron transport. Xu et al. (64) conducted a comparative study on the electronic properties of four DNA bases (A, C, G and T) using STM, finding that G exhibited the highest conductivity among the bases, whereas T displayed the lowest conductivity. This variation was attributed to each base’s ability to donate and accept electrons, which impacts how easily electrons could move through each base.

Hamers et al. (65) presented current imaging tunnelling spectroscopy, a spectroscopic technique that allows for I–V curves to be captured at individual points or specific locations while conducting a surface scan, resulting in spatially resolved I–V data. This approach allows for the compilation of a current map at any voltage within the range covered by the voltage sweep in the I–V curve, thereby facilitating the direct comparison of electronic details at any desired voltage with surface topography. Thus, through the examination of peaks in the derivatives of these spectra, one may pinpoint energy levels linked to high LDOS, such as surface states or resonances.

The I–V curves for the Oligo1 normal PINK1 DNA molecules were recorded at three regions, A1–C1, A2–C2 and A3–C3, each of which is characterised by different sequence homologies; moreover, the voltage gaps were significantly variable across these regions, with averages of 0.785 ± 0.317 V, 1.204 ± 0.198 V, and 0.341 ± 0.208 V, respectively. The highest V_g_ at A2–C2 correlates with the sequence heterogeneity of the area, suggesting a relationship between sequence variability and electronic behaviour. The Oligo2 mutant PINK1 DNA molecules exhibited altered electronic properties compared to their normal counterpart. The voltage gaps at points A1–C1, A2–C2 and A3–C3 were 0.626 ± 0.094 V, 0.676 ± 0.495 V and 0.873 ± 0.297 V, respectively, showing a relatively uniform distribution of electronic conductivity. These findings suggest that the mutation in PINK1 DNA molecules introduces changes in its electronic behaviour, particularly in regions of sequence difference.

Oligo1 normal and Oligo2 mutant PINK1 DNA molecules differ in the sequence at the mutation A2–C2 region. For normal DNA molecules, the mean V_g_ was 1.204 ± 0.198 V, whereas for mutant DNA molecules it was 0.676 ± 0.495 V. Statistical analysis using independent-sample *t*-tests confirmed that while the distributions were normal and variances homogenous, there was insufficient evidence to establish significant differences in mean V_g_ values at a *P* < 0.05 level. These results indicate that sequence mutations in PINK1 DNA molecules influence its electronic conductivity, particularly in nonidentical regions. This supports the hypothesis that DNA sequence alteration modulates DNA’s electronic properties (63).

## Conclusion

Our experiment reveals that the voltage gap between the synthetic Oligo1 normal and Oligo2 mutant PINK1 DNA molecules is not statistically different, suggesting that differences in DNA sequence alone may not directly alter the electrical properties of PINK1 DNA molecules in isolation. However, this result does not diminish the established role of PINK1 mutations in early-onset PD caused by mitochondrial dysfunction. We conclude that the effects of PINK1 mutations are manifested at the functional (protein) and organellar (mitochondrial) levels and not solely through the biophysical properties of DNA molecules. The convergence of mitochondrial failure, impaired protein interactions and compensatory mechanisms collectively drives PD pathology. Ultimately, the results of this work suggest that future studies prioritise research into therapies involving the PINK1-Parkin signalling pathway and mitochondrial health.

## Figures and Tables

**Table 1:** Comparison of the length *L* between the measured profile analyses with STM and the estimated size with Avogadro molecular modelling software for the Oligo1 normal and Oligo2 mutant PINK1 DNA. The length was measured on six different molecules of each DNA sample, as depicted in Figure 1.

	Length, *L* (nm)							

	Oligo1				Oligo2			

	STM			Avogadro	STM			Avogadro
	
		Median	Mean ± SD			Median	Mean ± SD	
*L* *_1_*	12.308	12.679	12.551 ± 0.304	5.332	8.762	8.570	8.531 ± 0.397	5.352
*L* *_2_*	12.704				8.383			
*L* *_3_*	12.769				8.452			
*L* *_4_*	12.051				9.028			
*L* *_5_*	12.821				7.873			
*L* *_6_*	12.653				8.687			

STM = Scanning tunnelling microscopy; SD = standard deviation

**Table 2:** Measurements of the voltage gaps on the Oligo1 normal and Oligo2 mutant PINK1 DNA molecules at points A1–C1, A2–C2 and A3–C3 in Figures 3A and 3B

Points	Voltage gap, V_g_ (V)					

	Oligo1			Oligo2		

		Median	Mean ± SD		Median	Mean ± SD
**A1**	0.576	0.628	0.785 ± 0.317	0.522	0.651	0.626 ± 0.094
**B1**	0.628			0.651		
**C1**	1.150			0.706		

**A2**	1.259	1.259	1.204 ± 0.198	0.733	0.733	0.676 ± 0.495
**B2**	1.368			0.154		
**C2**	0.983			1.140		

**A3**	0.579	0.252	0.341 ± 0.208	0.936	0.936	0.873 ± 0.297
**B3**	0.252			0.549		
**C3**	0.192			1.134		

SD = standard deviation

**Table 3:** The Shapiro–Wilk test for normality of the voltage gaps of the Oligo1 normal and Oligo2 mutant PINK1 DNA molecules, respectively, from Figures 3A and 3B, at points A1–C1, A2–C2 and A3–C3

Points		Descriptive statistics - Voltage gap (V)				Distribution normality test			

		*N*	Mean	SD	SEM	DF	*W*	*P*-value	Decision at *α* = 0.05
**A1–C1**	Oligo1	3	0.785	0.317	0.183	3	0.817	0.157	*P > α*, cannot reject *H_0_*, so the distribution is normal
	Oligo2	3	0.626	0.094	0.055	3	0.949	0.564	*P > α*, cannot reject *H_0_*, so the distribution is normal

**A2–C2**	Oligo1	3	1.203	0.198	0.115	3	0.941	0.531	*P > α*, cannot reject *H_0_*, so the distribution is normal
	Oligo2	3	0.676	0.495	0.286	3	0.990	0.808	*P > α*, cannot reject *H_0_*, so the distribution is normal

**A3–C3**	Oligo1	3	0.341	0.208	0.120	3	0.863	0.276	*P > α*, cannot reject *H_0_*, so the distribution is normal
	Oligo2	3	0.873	0.298	0.172	3	0.966	0.648	*P > α*, cannot reject *H_0_*, so the distribution is normal

DF = degree of freedom. In the Shapiro–Wilk test, DF = *N* (56); *H_0_* = null hypothesis; *N* = number of observations; *P* = probability; SD = standard deviation; SEM = standard error of the mean; *W* = Shapiro–Wilk statistic; *α* = significance level, where a threshold (*α ****=*** 0.05) is used to determine whether the *H_0_* is rejected. In the Shapiro–Wilk test, the *H_0_* refers to the distribution being normal

**Table 4:** The *F*-test for homogeneity of variance of the voltage gaps of the Oligo1 normal and Oligo2 mutant PINK1 DNA molecules, respectively, from Figures 3A and 3B, at points A1–C1, A2–C2, and A3–C3

Points		Descriptive statistics - Voltage gap (V)				Homogeneity of variance test				

		*N*	Mean	SD	s^2^	DF	*F* *_α/2, N_*_*_1_*_ *_– 1, N_*_*_2_*_ *_– 1_*	*F*	*P*-value	Decision at *α* = 0.05
**A1–C1**	Oligo1	3	0.785	0.317	0.101	2	19.000	11.297	0.163	*F <* critical value and *P > α*, cannot reject *H_0_*, insufficient evidence to infer that the two variances are significantly different
	Oligo2	3	0.626	0.094	0.009	2				
**A2–C2**	Oligo1	3	1.203	0.198	0.039	2	19.000	0.160	0.276	*F <* critical value and *P > α*, cannot reject *H_0_*, insufficient evidence to infer that the two variances are significantly different
	Oligo2	3	0.676	0.495	0.246	2				
**A3–C3**	Oligo1	3	0.341	0.208	0.043	2	19.000	0.490	0.658	*F <* critical value and *P > α*, cannot reject *H_0_*, insufficient evidence to infer that the two variances are significantly different
	Oligo2	3	0.873	0.298	0.089	2				

DF = degree of freedom; In the *F*-test, DF = N_x_–1 (58); *F* = *F*-test statistic; *F_α/2, N_*_*_1_*_*_– 1, N_*_*_2_*_*_– 1_* = critical value of the *F*-table for *α* = 0.05 (57); *H_0_* = null hypothesis; *N* = number of observations; *P* = probability; s^2^ = sample variance; SD = standard deviation; *α* = significance level, where a threshold (*α ****=*** 0.05) is used to determine whether the *H_0_* is rejected; In this *F*-test, the *H_0_* refers to the equal variances of the two samples, s_1_^2^ = s_2_^2^

**Table 5:** The independent-samples *t*-test for the voltage gaps of the Oligo1 normal and Oligo2 mutant PINK1 DNA molecules from Figures 3A–3B, at points A1–C1, A2–C2, and A3–C3

Points		Descriptive statistics – Voltage gap (V)					Independent-sample *t*-test				
		*N*	Mean (*χ̄*)	SD	SEM	Median	DF	*t* *_crit_*	*t*	*P*-value	Decision at *α* = 0.05
**A1–C1**	Oligo1	3	0.785	0.317	0.183	0.628	4	2.776	0.828	0.454	*t < t_crit_* and *P > α*, cannot reject *H_0_*, insufficient evidence to infer that *χ̄*_1_ is significantly different from *χ̄*_2_
	Oligo2	3	0.626	0.094	0.055	0.651					

**A2–C2**	Oligo1	3	1.203	0.198	0.115	1.259	4	2.776	1.712	0.162	*t < t_crit_* and *P > α*, cannot reject *H_0_*, insufficient evidence to infer that *χ̄*_1_ is significantly different from *χ̄*_2_
	Oligo2	3	0.676	0.495	0.286	0.733					

**A3–C3**	Oligo1	3	0.341	0.208	0.120	0.252	4	2.776	−2.537	0.064	*t < t_crit_* and *P > α*, cannot reject *H_0_*, insufficient evidence to infer that *χ̄*_1_ is significantly different from *χ̄*_2_
	Oligo2	3	0.873	0.298	0.172	0.936					

DF = degree of freedom; In the independent-sample *t*-test, DF = *N*_1_+*N*_2_–2 (54); *t_crit_* = critical value of the *t*-table for *α* = 0.05 (54); *t* = *t*-test statistic; *H_0_* = null hypothesis; *N* = number of observations; *P* = probability; SEM = standard error of the mean; SD = standard deviation; *α* = significance level, where a threshold (*α ****=*** 0.05) is used to determine whether the *H_0_* is rejected; In this independent-sample *t*-test, the *H_0_* refers to the equal means of the two samples, *χ̄*_1_ = *χ̄*_2_
